# Mesenchymal stem cell perspective: cell biology to clinical progress

**DOI:** 10.1038/s41536-019-0083-6

**Published:** 2019-12-02

**Authors:** Mark F. Pittenger, Dennis E. Discher, Bruno M. Péault, Donald G. Phinney, Joshua M. Hare, Arnold I. Caplan

**Affiliations:** 1 Department of Surgery, University of Maryland School of Medicine, 10S. Pine Street, Baltimore, MD 21212 USA; 2 Biophysical Engineering Labs, University of Pennsylvania, 129 Towne Bldg, Philadelphia, PA 19104-6393 USA; 3 Orthopedic Hospital Research Center, UCLA/Orthopedic Surgery, 615 Charles E. Young Drive, Los Angeles, CA 90095 USA; 4 MRC Centre for Regenerative Medicine, University of Edinburgh, 5 Little France Drive, Edinburgh, EH16 4UU UK; 5 Department of Molecular Medicine, A231, The Scripps Research Institute, 130 Scripps Way, Jupiter, FL 33458 USA; 6 Interdisciplinary Stem Cell Institute, University of Miami Miller School of Medicine, Biomedical Research Building/Room 908, PO Box 016960 (R-125), Miami, FL 33101 USA; 7 Department of Biology and Skeletal Research Center, Case-Western University, Millis Science Center, Room 118, 10900 Euclid Ave, Cleveland, OH 44106 USA

**Keywords:** Mesenchymal stem cells, Stem-cell research

## Abstract

The terms MSC and MSCs have become the preferred acronym to describe a cell and a cell population of multipotential stem/progenitor cells commonly referred to as mesenchymal stem cells, multipotential stromal cells, mesenchymal stromal cells, and mesenchymal progenitor cells. The MSCs can differentiate to important lineages under defined conditions in vitro and in limited situations after implantation in vivo. MSCs were isolated and described about 30 years ago and now there are over 55,000 publications on MSCs readily available. Here, we have focused on human MSCs whenever possible. The MSCs have broad anti-inflammatory and immune-modulatory properties. At present, these provide the greatest focus of human MSCs in clinical testing; however, the properties of cultured MSCs in vitro suggest they can have broader applications. The medical utility of MSCs continues to be investigated in over 950 clinical trials. There has been much progress in understanding MSCs over the years, and there is a strong foundation for future scientific research and clinical applications, but also some important questions remain to be answered. Developing further methods to understand and unlock MSC potential through intracellular and intercellular signaling, biomedical engineering, delivery methods and patient selection should all provide substantial advancements in the coming years and greater clinical opportunities. The expansive and growing field of MSC research is teaching us basic human cell biology as well as how to use this type of cell for cellular therapy in a variety of clinical settings, and while much promise is evident, careful new work is still needed.

## Introduction

MSCs have become widely studied over the past ~30 years for their interesting cell biology, broad-ranging clinical potential, and as a central building block in the rapidly growing field of tissue engineering. MSCs grow readily in the culture dish, have intrinsic differentiation potentials not found previously in other cells, and produce an abundance of useful growth factors and cytokines. The isolation of MSCs from various tissues and their re-implantation at other sites raises questions about the natural in vivo MSCs and their ability to normally repair endogenous tissues, a process that clearly diminishes with age. Mesenchymal cell replacement in the large numbers needed to treat significant tissue injury requires engraftment, structural organization and cellular differentiation—a complex process that has made much progress but remains unperfected. Friedenstein was first to culture bone-forming cells from guinea pig and Owen re-energized this inquiry by expanding such work to rats.^1,2^ The isolation and culture expansion of human bone marrow MSCs were reported in 1992 ^3^ and their infusion into patients was begun as early as 1993 as reported in 1995.^4^ Over the past 25 years the infusion procedures have exhibited an excellent safety profile, so much so that there are now over 950 registered MSC clinical trials listed with the FDA. There have been over 10,000 patients treated in a controlled clinical setting, of which 188 early trials (phase 1 or phase 2) have been completed and ten studies have advanced to phase 3 (Mesenchymal stem cells search at www.clinicaltrials.gov and https://celltrials.org/public-cells-data/msc-trials-2011-2018/65). Worldwide, for the years 2011−2018, there were 1043 MSC trials planned with a targeted enrollment of 47,548 patients (Mesenchymal stem cells search at www.clinicaltrials.gov and https://celltrials.org/public-cells-data/msc-trials-2011-2018/65). For comparison, bone marrow and hematopoietic stem cell (HSC) transplantations have been practiced since 1957, and through 1983, the first 25 years, about 9000 patients were treated.^5^

**Fig. 1:**
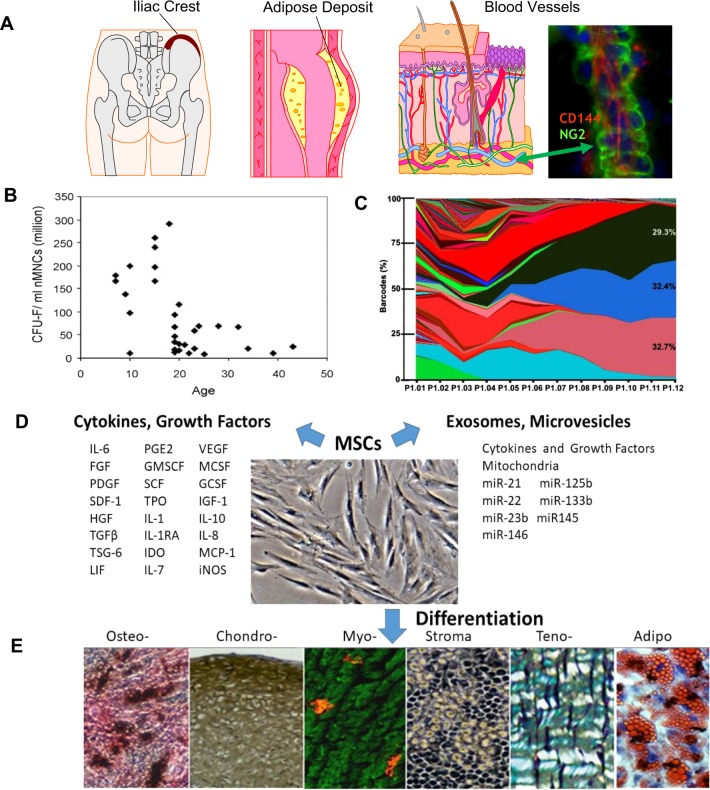
Characteristics of MSCs. **a** MSCs can be readily isolated from bone marrow and adipose tissue but all tissues harbor MSC-like cells as part of the microvasculature. **b** The number of MSCs, indicated here as colony-forming units (CFU-F), isolated from bone marrow drops off after 15–20 yrs of age and continues to decrease.^13^ **c** MSCs are rare in bone marrow and are culture-expanded to achieve high numbers for research or therapeutic use. However, there is a decrease in the clonal complexity with increased passaging,^23^ but the effect of this process on MSC uses is unclear. **d** The MSCs are known to produce a large number of soluble or vesicle-bound growth factors and cytokines, as well as microRNAs, that can signal to other cells and tissues. **e** The culture-expanded MSCs can differentiate to multiple cell lineages under separate specific in vitro conditions. The standard chrondro-, osteo- and adipo- differentiation conditions^6^ are widely used but additional in vitro conditions promote smooth muscle and striated muscle gene expression; changing medium conditions can induce expression of cardiac and liver genes. Once differentiated, the MSCs express virtually all the hallmark genes of the differentiated cell types. Currently, the more prominent MSC therapeutic uses take advantage of the MSC’s production of factors and the responsiveness of other interacting cells, such as cells of the immune system (see Table 1).

The most common and longest utilized adult source tissues for human MSCs are bone marrow^3,6^ and the adipose tissue stromal vascular fraction^7,8^ and these sources form the foundation for most of the data in this field (Fig. 1a). These are harvestable human tissues that are thought to be renewable (bone marrow) or unwanted (adipose). There are also two young “adult” tissues, umbilical cord tissue^9^ and placenta,^10,11^ that are excellent sources of human MSCs, and these tissues are normally discarded at birth. The decision to use autologous MSCs from bone marrow or adipose, or an allogeneic source tissue to isolate MSCs is a fundamental clinical decision, but both have shown success producing large numbers of MSCs.^6,8^ For example, a target dose of 100–150 million human MSCs can be produced from 25 ml of bone marrow by cell culturing in about 3 weeks and this number of packed cells has a volume of about 0.4−0.5 ml.^12^ There are still surprisingly few animal research reports or clinical studies that use autologous MSCs and most studies use allogeneic MSCs. In humans, there is also a recognized drop-off with age in the number of isolatable MSCs found in bone marrow, suggesting a very different set of circumstances in the aging population with their more injury-prone tissues than in young adults^13^ (Fig. 1b). In the sections below, we highlight developments and understanding in the cell biology of MSCs, a paradigm shift in their mechanism of action, and improvements in the clinical use of MSCs.

A prominent question is whether in vitro cultured MSCs represent any stage of natural in vivo MSCs, or similar cells found during development.^14^ It is useful to remember that the embryological development of mesenchymal tissues is complex and proceeds from both trunk and head (neural crest-derived) mesenchyme, and these two cell sources are interwoven in some tissues such as the heart. Within a developing organism, cellular differentiation would appear to be deterministic: we can predict the fate of similar cells in the next offspring or all offspring of future generations, or even in distant or unrelated species. However, we also know from many studies in embryology that cells from one presumptive tissue can be implanted in another tissue and acquire a different fate. Their fate is locally regulated by the new environment and the further development of the implanted cells is selective and not directive. This is a feature and not a flaw of stem/progenitor cells such as MSCs. The early human developmental biology of mesenchymal tissues represents a very specific series of temporal events^14^ and is far removed from the tissue repair that occurs in the adult 15−80 years later, and while early development might have something to teach us about using cultured MSCs for repairing and regenerating adult tissues, at this time it is unknown exactly what that will be.

## The unfortunate rise of unregulated stem cell clinics

MSCs and other stem cells offer remarkable potential but our understanding of their science and medical applications are not ready for unregulated, widespread use. The complexity of tissue repair and cell replacement makes it clear that the proliferation of questionable “stem cell clinics” and off-shore medical tourism offices promoting their autologous “stem cell treatments” of unknown and unproven efficacy will not solve patient maladies in a meaningful way. The divergence between reputable clinical trials and the premature marketing of stem cell products to the public has broadened the gap, and led to confusion in the press as highlighted by Galipeau et al.^15^ There are over 700 clinics offering direct-to-consumer marketing of “stem cell” treatments.^16^ We cannot support or recommend any treatment utilizing MSCs that does not use characterized cell product, maintain accurate records, measure intermediate parameters, predetermined surrogate endpoints and track and report final patient outcome(s). These steps are common practice in FDA registered trials, but too demanding for under- or unregulated clinics. A recent study (see Murray et al.^16^ reference for details) has offered a consensus report on the parameters needed to improve cell therapy outcomes for both patients and practitioners using the acronym DOSES: D—Donor, O—Origin tissue, S—Separation Method, E—Exhibited Characteristics, S—Site of Delivery.

## MSC cell biology—microheterogeneity, temporal stochasticity and diversity at the single cell level

The defining characteristics of a vertebrate stem cell are the ability to divide (symmetrically or asymmetrically), to be motile, to differentiate to multiple lineages and to become organized into multifunctional groupings. To become functionally organized, stem cells need a permissive and instructive environment. Hence, phenotypic reprogramming of stem cells is dependent on the cellular environment and the temporal application of instructive agents and their persistence. This attribute is exemplified in MSCs by their osteo-, adipo-, or chondro-genic differentiation^6^ over 1−3 weeks (Fig. 1e) but further illustrated by the stepwise acquisition of cardiomyocyte properties by sequentially changing the culture conditions over 3−4 weeks as shown by Terzic and colleagues.^17^ These population differentiation outcomes of MSCs are a composite result and reflect properties at the single-cell level, but the timing of events for each cell may vary somewhat. It has been recognized that stem cell populations are not homogeneous but rather the cells therein often behave as individual cells^18^—even if they are clonally derived.^19^ This temporal stochasticity is a common feature of stem/progenitor cells and occurs throughout development.^20^ The stochastic events and processes of stem/progenitor cells are perhaps the most difficult to model or approach experimentally, but we can see similar events in vitro.^21,22^ In the case of MSCs, a single cell may enter a phase of repeated cell division to create a population containing millions of cells, or die by apoptosis in response to nutrient deprivation, DNA damage, membrane injury etc. For example, when MSCs in culture are labeled with lentivirus vectors encoding individual tags to trace the fate of daughter cells, stochastic processes cause the loss of some clones and the proliferation of others, such that a cultured MSC population with an initial complexity of 70 is reduced to a complexity of 3 to 4 surviving clones, and these resulting clones do not represent the most abundant clones at the start^23^ (see Fig. 1c). While troubling our view of culture-expanded MSCs as a homogeneous population, if all the remaining cell clones have the same stereotypical behavior or “abilities” as the starting cells, this reduction in MSC population complexity may not result in the loss of potential or utility. Studies to understand how these events alter adult MSCs and MSC population composition and function, with respect to both their stem/progenitor and paracrine activities, in vitro and in vivo, are important for our biological and clinical potential understanding. Studies of clonal activities of HSCs have long indicated the rise and fall of individual clones, but this may not impact their functional or clinical outcomes.^24,25^ For in vivo intestinal epithelial stem cells too, the clonal expansion/extinction process has strong experimental evidence.^26^ Recent studies indicate that growth factors, cytokines, and other bioactive factors produced by MSCs may be contained in exosomes and microvesicles that function in a paracrine manner.^27–30^ Although the role of exosomes and microvesicles in normal MSC physiology and as therapeutic entities is emerging, how the exosome/microvesicle production and composition are influenced by stochastic processes, clonal expansion and culture complexity or MSC differentiation remains largely unexplored.

## MSC transcriptome and phenomics

MSCs are still largely defined by their in vitro expression of a restricted subset of cell surface proteins, and their capacity for stimulus-induced tri-lineage differentiation.^6,31^ While this minimalistic definition has sufficed for almost two decades and is still widely used today, the field should benefit from the large repository of gene expression-based data (GEO Datasets) that interrogates the MSC transcriptome, and the important expression changes that occur following culture expansion, hypoxia preconditioning, stimulus-directed differentiation, trans-differentiation, exposure to biologics, and coculture with other cell types. The genome-wide gene expression studies can provide insight into the biological nature of MSCs, their expected physiological function, role in disease pathophysiology, and probable therapeutic mode of action. Understanding MSC gene expression data holds promise for refining the operational definition of MSCs, clarifying their native physiological function, and informing how culture conditions and clinical manufacturing protocols can best characterize their composition and function prior to patient administration.

MSC gene expression studies were initially focused on establishing an identity for bone marrow-derived MSCs (BM-MSCs) in vitro that could be shared across laboratories. Toward this goal, the transcriptome of human and mouse BM-MSCs was cataloged via serial analysis of gene expression (SAGE),^32^ and the nature of the catalogued transcripts were shown to reflect their stem/progenitor properties and paracrine activities related to hematopoiesis support and skeletal homeostasis. Along with related cellular studies, the gene expression data support the skeletogenic, angiogenic, anti-inflammatory and immunomodulatory activities of MSC populations that are widely utilized for clinical therapies today. Additionally, studies have shown that MSCs isolated and cultured from different tissues/organs are more closely related to each other than other mesodermal lineages, and that their phenotypic signature is similar to that of perivascular cells,^33,34^ providing a physiological basis for the widespread anatomical distribution of MSCs or MSC-like cells in vivo (see below.) The extent to which the MSC gene expression is influenced by their culture conditions and can be manipulated remains important for their clinical utility.

Recent RNA-seq studies have furthered our understanding of how MSCs respond at the cellular level to differentiation-inducing stimuli. For example, one such study identified prominent changes in the MSC transcriptome following differentiation to the adipogenic vs. osteogenic lineage, and ChIP-Seq studies revealed that the epigenome of MSC-derived osteoblasts, but not adipocytes, more closely resembled that of naïve cultured MSCs.^35^ The MSC genome was also shown to contain a high degree of overlap for binding sites of master transcriptional regulators, such as RUNX2 and C/EBPβ, that are epigenetically reduced in size following differentiation, and these promoter regions exhibited high plasticity that enabled MSCs to trans-differentiate from adipocytes to osteoblasts and vice versa.^36^ These transcriptional pathways may be relevant to the in vivo differentiation fate of MSCs.^37^ The Wnt intracellular signaling protein (WISP-1 or CCN4) was recently shown to modulate the osteo- and adipogenic lineages.^38^ These findings broaden the concept of in vitro lineage priming used initially to provide the molecular basis for MSC multi-potency^39^ and may yield improved therapies.

In addition to differentiation-inducing stimuli, other treatments have also been identified that alter the biological activity of MSCs by altering gene expression. For example, it was established early on that rodent MSCs exhibit enhanced growth and osteogenic potential under low oxygen (5%) levels,^40–42^ which mimics conditions in the bone marrow niche. Subsequently, it was shown that transient exposure of MSCs to hypoxic conditions (<2% oxygen saturation) enhances their proangiogenic activity in vitro^43–45^ and in vivo^46–48^ and positively impacts growth and survival.^49–51^ Profiling studies have revealed that hypoxic preconditioning significantly alters expression of a small subset of genes linked to cell proliferation and survival, glycolysis, and vasculogenesis/angiogenesis in MSCs, the majority of which were upregulated.^52,53^ A similar approach is being used to interrogate how MSCs respond to inflammatory stimuli to gain further insight into their anti-inflammatory and immunomodulatory activities. For example, in vitro stimulation of human MSCs with lipopolysaccharides, a ligand for TRL4, induced expression of transcripts involved in chemotaxis and inflammatory responses that were principally orchestrated by interferon regulatory factor (IRF1) and nuclear factor kappa B (NF-κB).^54^ Alternatively, exposure to interferon (IFN)-gamma licenses the immunosuppressive activity of MSCs by inducing expression of indoleamine 2,3-dioxygenase (IDO1), an enzyme in the kynurenin pathway that consumes tryptophan^55,56^ resulting in reduced inflammation, and stimulation by TNF upregulates expression of the anti-inflammatory protein TSG-6.^57^ Importantly, while TNF- and IFN-gamma-stimulated MSCs expressed distinct sets of proinflammatory factors, these proteins were shown to function synergistically to uniformly polarize MSCs toward a Th1 phenotype characterized by expression of the immunosuppressive factors IL-4, IL-10, CD274/PD-L1 and IDO.^58^ This finding is also significant because at the population level, hierarchical clustering of gene expression data from MSC donors revealed that nonstimulated populations exhibited a significantly greater degree of inter-donor heterogeneity.^59^ Therefore, treating MSCs with potent stimuli has a normalizing effect on the population and may largely erase interdonor differences in MSC function, and this “cytokine priming” should be tested in animal and clinical studies.

It is important to point out that in addition to enhancing paracrine signaling, exposure to inflammatory stimuli such as TNF and IFN-gamma produces other notable effects on MSCs. For example, IFN-gamma upregulates expression of genes associated with programmed cell death and cellular apoptosis, reflecting its profound negative impact on MSC growth and survival.^60^ Such gene expression responses of MSCs to IFN-gamma treatment are also accompanied by alterations in gross morphology.^61^ MSCs induced toward the osteogenic lineage have been shown to exhibit upregulation of IFN-gamma inducible genes^62,63^ and a concomitant impairment in angiogenic activity.^64^ Similarly, analysis of MSCs from TSG-6 knockout mice revealed profound alterations in cell morphology, impaired growth, and loss of tri-lineage differentiation potential.^65^ In this study, RNA-seq analysis identified 1537 downregulated and 1487 upregulated genes in TSG-6 null MSCs as compared to wild-type cells, and these genes mapped to biological processes including cell cycle, cell death and survival, cell morphology, cellular movement, DNA replication and repair. Notably, the expression of several transcription factors involved in regulating cell division, stem cell differentiation, and Wnt signaling was found to be inhibited upon TSG-6 deletion. These data are consistent with other studies showing that pathways controlling cell growth, differentiation, and paracrine signaling are mechanistically linked, and in some cases may be mutually exclusive. For example, a recent study showed that treatment of equine adipose-derived stromal cells with interleukin-1β and/or TNF compromised their tenogenic properties in part by reducing expression of the tenogenic transcription factor scleraxis.^66^ Consequently, more studies are needed to predict which culture conditions are best suited for a desired cellular response or particular clinical indication.

Is there a profile of expressed genes that defines MSCs and can be used to predict success in their therapeutic use? A large integrative analysis of existing MSC genomic datasets was used to establish an “MSC classifier” that accurately distinguishes MSC from non-MSC samples with over 97% accuracy.^67^ The gene expression and protein data that contribute to the MSC classifier can add rigor to the current definition of MSCs. Similarly, a comparative genomics approach was recently used to develop a “Clinical Indications Prediction” (CLIP) scale based on in vitro TWIST1 expression levels that reveals differences in the biological activity of different donor MSCs. The CLIP scale may have utility for matching the biological activity of MSC donor populations to specific disease indications and may thereby improve outcomes of MSC-based preclinical models of disease and subsequent clinical studies. This approach could also interrogate in real time how preconditioning regimens, cytokine priming and manufacturing processes may improve the predictability of MSC biological activity and clinical outcomes. The reproducibility of MSC isolation and gene expression is further supported by examining multiple isolations of bone marrow MSCs and comparing results to HSCs, NSCs and ESCs, wherein it was found that the MSCs clustered together more than the other stem cell types tested.^68^ The role(s) of microRNAs and circular RNAs in sustaining MSC identity are likely to be another important distinguishing indicator of phenotype.^69^ A broad analysis of expressed genes by RNA deep sequencing and translated proteins by nano-liquid chromatography MS/MS for both BM-MSCs and ESC-derived MSCs found many similarities and suggested new membrane surface proteins that may be useful for phenotypic identification in future studies.^70^

Keep in mind that for therapeutic MSC products, a release assay related to the clinical proposed function of the MSCs in vivo is requested by the FDA at phase 1 and is required by phase 3. Consequently, these RNA-Seq and ChIP databases and related resources provide a tool kit that should be more thoroughly tested to match appropriate donor MSCs, manufacturing protocols, and patients to improve response rates. This means that MSCs will be produced as a focused product for each therapeutic application and require more specific release criteria and likely include a population gene expression measure such as the TWIST-based CLIP assay as well as an individual cell measure such as flow cytometry. For MSCs for the treatment of graft vs. host disease (GVHD), the release assay should reflect the anti-inflammatory activity of the MSC product^71^ and an inexpensive and accessible flow cytometry assay such as that of Ribeiro et al.^72^ that can quickly test >10,000 individual MSCs in minutes gives a readout of both the single-cell analysis and a population (product) assay. But if, however, the therapeutic mode is found to be not the cell, but a secreted cytokine(s), exosome enclosed miRNA or factor(s), then more specific assay(s) will be required. Recently, Kaushal and colleagues were able to demonstrate that, following injection into heart tissues, the expanded cardiac-derived cell population (cardiac progenitor cells or CPCs) that includes an MSC-like population, alter their beneficial exosome expression in the in vivo setting.^30^ Further understanding of the localized response(s) of ex vivo expanded progenitor cells placed into the in vivo damaged tissue setting is needed for the therapeutic development of cellular therapies.

## MSCs and related vascular cell types

Despite extensive efforts to characterize MSCs, these remain, fundamentally, a product of their extended cell culture conditions. They originate from tissue but are they “real” stem cells in vivo? Does the tissue dissociation, adhesion to tissue culture plastic and growth in serum-supplemented medium isolate and drive the sustained proliferation of a rare, elusive tissue-residing progenitor cell(s), or is it that our tissue culture acumen has produced a valuable “artifact” of the process? Some studies may have equated the MSC to a previous histologically identified cell in bone marrow such as the reticulocyte, Weston-Bainton cell, a stromal cell, a fibroblast, etc., but the rare nature of MSCs makes this unlikely and the in vivo identity(s) of MSCs remains obscure—despite the now broad use of MSCs in tissue engineering and regenerative medicine. Further, although bone marrow was first used for MSC isolation and considered a renewable source, MSC-like cells have been isolated from many tissue sources including harvested adipose tissue,^7,8^ umbilical Wharton’s jelly,^9^ placenta,^10,11^ skin^73^ and the roots of shed teeth.^74^ The studies of microvascular pericytes soon overlapped with attempts to uncover the innate identity of in vivo MSCs and indicated phenotypic similarities between the two,^75–77^ and Crisan et al.^78^ showed that pericytes purified by flow cytometry from diverse human organs and cultured for several passages are indistinguishable from conventional, bone marrow-derived MSCs in terms of morphology, proliferation kinetics, surface antigen expression, and differentiation potential, in vitro and in vivo. Moreover, the canonical MSC surface marker combination of CD44^+^/CD73^+^/CD90^+^/CD105^+^ is detectable on pericytes in situ^78^. This paradigm was later extended to perivascular spaces around larger arteries and veins, in which the outermost tunica adventitia contains a population of fibroblast like presumptive MSCs.^79^ The presence of similar cells around the microvasculature is being presently investigated. This affiliation with the vasculature puts MSCs in position to respond quickly to tissue damage. To further understand the respective potential of pericytes and adventitial progenitors, the transcriptome of single cells sorted from human adipose tissue was determined and the differential gene expression, principal component and clustering analysis, as well as the construction of gene coregulation networks showed that adventitial cells constitute a more “primitive” population, expressing genes associated with “stemness”, such as nanog, c-myc, klf2, -4, -6, and osteogenic commitment and differentiation (runx2, nox4, notch2).^80^ Conversely, the pericytes appeared overall as more differentiated cells, expressing genes involved in angiogenesis and smooth muscle cell function (angpt2, acta2), in agreement with the in vivo function of these cells.

Accordingly, recent studies have revealed that in the course of osseous regeneration in vivo, pericytes principally stimulate neoangiogenesis, while adventitial cells are more directly involved in bone formation.^81^ When cultured in vitro, both perivascular cell types clearly establish an MSC population, but the respective contributions of pericytes and adventitial cells to the multipassage cultured MSC remain to be elucidated.

A largely unanswered question raised by the prospective identification of perivascular cells as innate MSC forerunners is whether these cells play the same progenitor role in their in vivo environment. Not surprisingly, RNA-Seq studies performed on human pericytes and adventitial perivascular cells before and after culture revealed dramatic differences in gene expression associated with their establishment in culture and the transition to the in vitro MSC phenotype, with up to one third of all expressed genes being significantly up- or downregulated. (Hardy et al., manuscript in preparation). This may suggest that perivascular cell -derived MSCs are profoundly modified, or even entirely initiated, by cell culture; however, cell lineage tracking in reporter transgenic mice has uncovered roles for pericytes as mesenchymal progenitors, in the adult, for white adipocytes,^82^ myoblasts,^83^ follicular dendritic cells,^84^ and profibrotic myofibroblasts,^85–89^ and both pericytes and adventitial progenitor cells are involved in the turnover and repair of dental tissues.^90^ A recent study confirming the MSC potential of mouse perivascular adventitial cells also uncovered a pathologic correlation and demonstrated that adventitial cells directly contribute to atheroma formation and calcification in remodeling large vessels, by differentiating into smooth muscle cells and osteoblasts, respectively.^91^ Thus, the modern version of embryological tissue transplantation suggests the MSCs have multipotentiality in vivo as well as in vitro.

However, considering the hundreds of billions of pericytes associated with the 50,000 miles of capillaries present in the human body—plus the other blood vessels—and even though no more than one in ten perivascular cells yields MSCs in culture, the global efficacy of the system to repair/regenerate tissues in the living adult organism would appear to be surprisingly low.^92,93^ The observed discrepancy between the robust potential exhibited by in vitro cultured MSCs from perivascular tissue and the modest endogenous role evidenced in vivo calls for investigations regarding the clonal selection and gene expression alterations that accompany their establishment in vitro to perhaps understand and facilitate the molecular control of their reprogramming into stem-like reparative cells in situ. It may also be that the perivascular MSCs are engaged in an important tissue function and are not available for mobilization. Once relieved of this obligation by tissue harvest and in vitro culture, the vascular derived MSCs seem free to pursue other roles. In the kidney, pericytes play diverse roles as mesangial cells in glomeruli and renin secreting cells in afferent arterioles. Yet, these specialized pericytes yield “MSCs” when purified, cultured and evaluated.^94^

## MSC responses—outside-in signaling on hard vs. soft substrates

The bone marrow MSCs reside in their in vivo niches where cell−cell interactions involving N-cadherins are thought to be key to maintaining the stem cell state with the essential interacting domains involving the peptide His-Ala-Val-Asp. As MSCs move away from this nurturing niche environment, they may encounter fewer cell−cell interactions and more extracellular matrix interactions. Most types of in vitro cultured adherent cells, including MSCs, assemble integrin-based focal adhesions that engage extracellular matrix molecules (fibronectin, laminins and collagens, initially supplied in vitro from serum) and form extensive cytoskeletal networks on rigid plastic or glass surfaces but not flexible substrates.^95^ In vitro cell culture typically utilizes negatively charged polystyrene dishes or flasks that aid attachment of extracellular matrix proteins and cells. However, for multipotential cells such as MSCs, these hard plastics may not be ideal for deciphering cell lineage potentials. MSCs grown on the rigid polystyrene culture surface can be directed to specific lineages—osteo-, chrondro- and adipogenic but only limited myogenic differentiation occurs (~1–2% positive for desmin and myosin heavy chain proteins). However, several days in culture on compliant substrates caused MSCs to exhibit additional characteristics consistent with the soft-tissue myo- and neuro- lineages.^96^

Tissues exhibit a range of stiffness in vivo, as quantified by the elastic modulus (Young’s modulus) measured over a range of 0.1–100 kPa. Brain and marrow tissue stiffness is about ~0.3 kPa, fat ~3 kPa, muscle ~10 kPa, and precalcified bone ~100 kPa, whereas tissue culture plastic or glass is much harder at »1000 kPa. MSCs in bone marrow can exhibit dendritic shapes and express some neuro-typical markers such as CD271, TRK A, B and C mRNAs, and a moderate fraction of MSCs cultured on soft gels that mimic the softness of marrow or neural tissue favor expression of nestin, β3 tubulin, and other neuro-typic traits.^96,97^ However, adipogenesis is a definitive soft-tissue differentiation pathway of MSCs and soft gels have been repeatedly found to be conducive to adipogenesis when compared to differentiation on stiff substrates, and stiff 2D substrates strongly favor osteogenesis, which is relevant to the epitaxial growth of bone, and similar findings have been reported for 3D gels.^98–104^ Growing MSCs for a longer time on either a soft or a stiff substrate progressively commits the cells to the corresponding lineages, making them refractory to rapid lineage switching by both soluble factors and substrate changes.^96,99^ The molecular basis for this commitment—be it DNA methylation, microRNA, soluble factors, or other—remains to be identified. With regards to the perivascular localization of in vivo MSCs discussed previously, it was recently demonstrated that purified human pericytes also can be induced to differentiate in culture into either osteocytes, chondrocytes or even neuron-like cells by modifying the stiffness of a hydrogel substrate.^101^

**Fig. 2:**
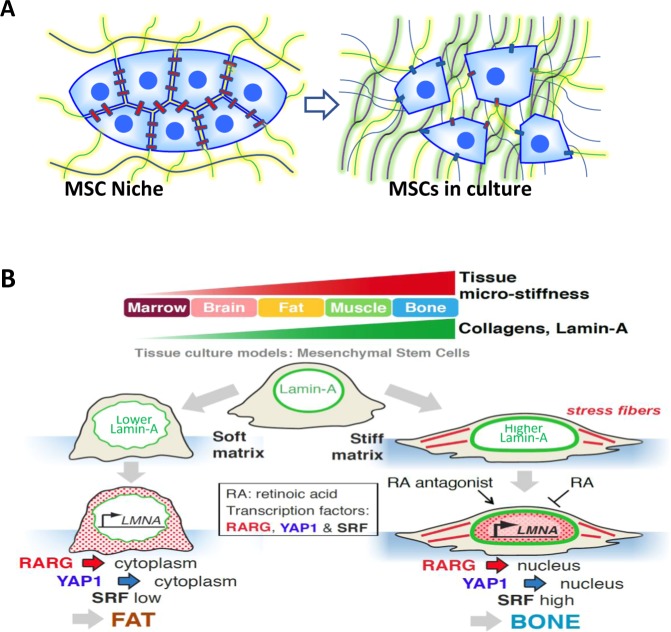
MSC interactions with cytoskeletal elements, cell−cell contacts, extracellular matrix and topography can have profound effects on multipotential MSCs. **a** Harvesting MSCs from a bone marrow niche with its condensed cell-rich environment and culturing them in vitro removes the cell−cell cadherin and connexin connections and replaces them with cell−substrate and cell−matrix interactions, as the cells produce more extracellular matrix. **b** The stiffness of the MSC culture surface and the nature of the environment have significant input to alter gene transcription and biological responsiveness of the MSCs through nuclear Lamin-A^104^ and YAP1^98^ (**b**) and the surface curvature can transduce cytoskeletal influence over MSC potential.^107^ Also, dynamic stretching and 3D matrix materials can provide new approaches to understanding MSC responses and potential therapeutic applications.

The molecular mechanisms that MSCs use to transduce the compliance of a matrix to lineage differentiation cues continue to be studied, with certain reproducible pathways emerging (Fig. 2). For MSCs, acto-myosin motility is more prominent on stiffer substrates when substrate adhesion is not limiting (too little or too much). Cytoskeletal contraction forces clearly contribute to differentiation, with the higher forces exerted on stiffer substrates favoring a stiff tissue (bone) lineage.^96,100^ Smooth muscle actin (SMA) assembles into high tension stress fibers and is understandably upregulated in MSCs on stiff substrates, and although SMA expression can be quite variable between MSCs even on a homogeneously stiff substrate,^102^ SMA does contribute to osteogenesis of MSCs.^105^ At the nuclear membrane, the structural protein lamin-A engages cytoskeletal stress fibers via linkage proteins that span the nuclear envelope, and high levels of lamin-A in MSCs favor osteogenesis whereas low levels favor adipogenesis, consistent with observations that lamin-A is high in stiff tissues but relatively low in soft tissues.^102,104^ The transcriptional co-activators YAP and TAZ primarily translocate (see Fig. 2b) into the nucleus in cells on stiff substrates to promote expression of differentiation genes for “stiff lineages”.^98,106^

Further, MSCs respond to the surface curvature of their substrate. When present on a rigid convex curvature of ~500 micron radius, the MSC’s long axis (~200 micron) is extended, the nucleus is flattened/deformed by stress fibers, there is more nuclear lamin-A and the cell is prone to osteogenic differentiation, whereas when the MSC is present on a concave surface of the same radius, the cell is more motile, has fewer stress fibers, the nucleus has greater curvature, less lamin-A and is “suspended” by the cytoskeleton.^107^ The foregoing description suggests a clonal group of MSCs may be induced to differentiate to several lineages in response to their migration over substrates of different compliance or curvature as may be found during development. Additionally, Fisher and colleagues recently demonstrated that MSC attachment followed by dynamic substrate movement (stretching) provides one more step in the “education” that MSCs may experience in vivo.^108^ The growth of MSCs on soft materials may also preserve the regenerative properties of the cells into later passages or allow them to recover from tissue culture-related aging.^109^ The biomaterials-MSC field is actively producing new data, especially in relation to 3D tissue mimetics which may have a profound effect on MSCs, and these data are needed for many growing in vivo applications.^110,111^ Adoption of reproducible methods for culturing MSCs on soft materials or dynamic substrates may provide greater therapeutic potential and effectivity. Can we then use the knowledge of MSCs responsiveness to culture substrate compliance, curvature, and micromovement to better prepare them for clinical applications?

## MSC paradigm shift—cell replacement to paracrine provider

The early demonstrated multipotential differentiation of MSCs fueled prospects for cell replacement where damaged tissue could be readily renewed. However, resolution of adult tissue damage wherein ounces of complex tissue must be dissolved, resorbed, renewed and remodeled, is a complex process not likely solved by the MSC itself. Over the past decade the emphasis has shifted toward harnessing the MSCs’ ability to produce factors and cytokines that stimulate innate tissue repair and modulate inflammation and immune responses (Table 1). Many MSC clinical trials are testing how the paracrine activity of these cells can be utilized, not the cells ability to differentiate to mesenchymal lineages. This is a very different mode of action from that seen with HSCs and their transplantation, a model that perhaps has hampered more than helped our understanding of MSCs. To date, the clinical trials with MSCs have established a strong safety profile and some success in patient subgroups has been evident (see below), and the MSC paracrine activities have fostered their examination in many diverse therapeutic applications. The immune modulation and anti-inflammation applications of MSCs are broadly applicable in damaged tissue, and the shift in emphasis from cell replacement to modifying the body’s cell and tissue responses from a clinical perspective reflects our progress in understanding the available ex vivo expanded MSCs. To what extent the culture-expanded MSCs reflect the endogenous adult tissue-resident MSCs is not yet clear, as discussed above.

**Table 1:** MSCs express many surface markers and secrete many proteins, immune modulating molecules and microRNAs.

Surface markers and cytokine receptors	Secreted growth factors and cytokines	Immunomodul-atory molecules	Micro RNAs
CD9	VEGF	TGF	miRNA-9-5p
CD44 HA Rec	FGF2	HGF	miRNA-10a
CD54 ICAM-1	FLT-3 Ligand	PGE2 antibac too	miRNA-10b
CD58 LFA-3	M-CSF	IL-1RA	miRNA-21
CD62L L-Selectin	G-CSF	IL-6	miRNA-23b
CD71 Transferrin Rec	GM-CSF inducible	IL10	miRNA-24
CD73 Ectonucleotidase	SCF	LIF	miRNA-29
CD90 Thy-1	LIF	HLA-G	miRNA-125b
CD105 Endoglin		IDO	miRNA-133b
CD106 VCAM-1		iNOS	miRNA-143-3p
CD117 KIT		TSG-6	miRNA-145
CDw119 IFNγR		Gal-1	miRNA-146b
CD120a TNFIR		Gal-9	miRNA-191-5p
CD120b TNFIIR		HO-1	miRNA-199
CD140b PDGFRB		LL37 antibac pep	let-7a-5p
CD146 MCAM			miRNA-222-3p
CD166 IGF1R			miRNA-451
CD221 IGF1R	**Integrins-positive**		miRNA-486-5p
CD222 IGF2R	CD49a α1		miRNA-1224
CD331 FGFR1	CD49b α2		
CD332 FGFR2	CD49c α3	**Integrins-negative**	**Hemato-negative**
SSEA-3	CD49e αv	CD11a αL	CD4
SSEA-4	CD51 aα	CD18 Cβ2	CD11b
HLA Class I	CD29 β1	CD49d α4	CD14
HLA-G	CD61 β3		CD34
(HLA Class II -inducible)	CD104 β4		CD45

Surface receptors are one of the ways MSCs respond to their surroundings and interact with other cells and tissue. Similar to the gene expression alteration under MSC differentiation conditions, some factors may be induced under certain conditions. This list is compiled data from human MSCs and is very similar in other species but may not be identical. Due to the large number of factors and publications, the reader should search PubMed for species MSCs and the factor of interest to find 10−50 references—the early work as well as the most recent

## MSC modulation of the immune system

Initially, early studies on MSCs envisioned autologous cell therapy for the orthopedic applications of bone and cartilage repair, and separate studies sought to provide “stromal” cytokine enhancement of bone marrow transplantation in cancer patients. The orthopedic studies initially flourished and animal studies looked very promising with autologous MSCs, and human clinical trials were planned. However, at least two findings prompted the testing of allogeneic MSCs in orthopedics, bone marrow transplantation, and also cardiac infarcts: (1) the costs to produce autologous MSCs for injection were substantial when it was understood that each patient’s culture-expanded MSCs would need to undergo extensive safety testing before infusion to assure the expansion process did not introduce any bacteria, viruses, etc. and (2) many patients previously treated for hematopoietic malignancies had diminished MSC numbers in their bone marrow, and the required autologous MSC dose could not be achieved as quickly as needed (2−3 weeks) to treat these patients.^112^ To address the second problem, allogeneic MSCs were isolated from an immunologically matched donor, a family member. These donors were not identical matches and it was anticipated that the use of these allo-MSCs would produce greater graft vs. host disease in the recipients—especially when the third-party HSC treatment was not a perfect match either. However, the investigators found LESS graft vs. host disease in the recipients, not more.^112,113^ This unexpected important medical benefit of allogeneic MSC treatment has been explored extensively to date. This also appeared to “solve” the other problem of cost by allowing large numbers of MSCs to be grown from a donor, and extensively tested, and then used to treat many patients, thereby reducing treatment costs.

**Fig. 3:**
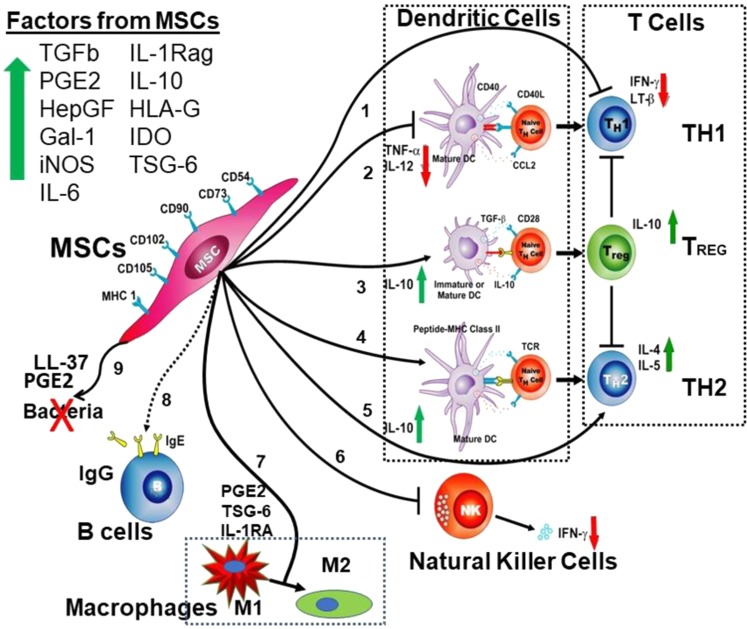
MSC—Immune cell interactions. Initial studies envisioned autologous use of MSCs. However, studies with immune cells demonstrated that MSCs are not immediately rejected by T cells and other immune cells, prompting the study of allogeneic MSCs in mutiple therapies. MSCs produce at least 11 factors known to affect immune cells. When interacting with T cells (pathways 1 and 5) MSCs cause a reduction in inflammatory T H1 and an increase in T Regs and T H2 cells with the concomitant decrease in IFNγ, increase in IL-10, IL-4 and IL-5. When MSCs interact with dendritic cells (pathways 2, 3,and 4) there is a decrease in proinflammatory mature DC1 with a decrease in TNF-α and IL-12, and an increase in immature DC and DC2, with increased expression of IL-10. When MSCs interact with natural killer cells (pathway 6) there is a decrease in the expression of IFNγ. When macrophages interact with MSCs (pathway 7), there is a decrease in the proinflammatory M1 phenotype and an increase in the anti-inflammatory M2 phenotype, with increased PGE2, TSG-6 and IL-1RA. MSCs can also reduce the secretion of antibodies from B cells (pathway 8) and inhibit bacterial growth by a direct or indirect mechanism (pathway 9). This figure is used with permission from Blood/Aggarwal and Pittenger^116^ and has been updated/modified from its original form.

Contemporaneous with early MSC/hematopoietic stem cell transplant clinical studies, in vitro studies tested allo-MSCs in mixed donor lymphocyte reactions and revealed the MSCs prevented lymphocyte proliferation, and do not cause apoptosis of T cells, rather the T cells will respond to subsequent lymphocyte challenge when the MSCs are removed.^114,115^ Many subsequent studies confirmed these findings. For cell−cell interaction, it was found that MSCs normally express major histocompatibility (MHC) Class I antigens on their surface and not Class II, but Class II antigens are upregulated by inflammatory agents. The intensive search for soluble factors secreted from MSCs that cause them to be immune-modulatory (Fig. 3) identified multiple factors^113,116–124^ that limit immune cell responses including transforming growth factor β, hepatocyte growth factor, prostaglandin E2, interleukin-10, interleukin-1 receptor antagonist, interleukin-6, human leukocyte antigen-G, leukocyte inhibitory factor, indoleamine-2, 3-dioxygenase, nitric oxide, galectins-1 and -9, and TNFα stimulated gene 6 (TSG-6). Further, MSCs skewed maturing immune cell populations resulting in increased regulatory T cells (T_Reg_), anti-inflammatory T_H_2 cells, and dendritic DC2 cells while fewer proinflammatory T_H_1 cells, dendritic DC1 cells, and fewer NK cells were found. MSCs also induced M1 macrophages to the anti-inflammatory M2 form and reduced IgG production from B cells. While many of these identified factors have been used individually to inhibit immune responses, the MSCs produce a more complete immune modulation owing to the multiple factors acting in unison.

This downmodulation of immune cell proliferation would seem to put the recipient at risk for higher infection rates but this is not seen in vivo when patients receive MSC infusions. The MSC’s production of antibacterial agents PGE2^116,125^ and LL-37 peptide,^126^ that may work in vivo through effects on hematopoietic cells, are at least part of the reason. Thus, the MSCs have been shown capable of modulating immune responses in situations where T, DC, macrophage and NK cell proliferation could lead to a runaway cytokine storm. This property of MSCs is highly desirable and is reflected in the many clinical trials which are testing the immune-modulatory and anti-inflammatory properties of MSCs.

Graft versus host disease (GVHD), a common complication following bone marrow or cord cell transplantation, represents a response of the developing new hematopoietic and immune system against the recipient host and can result in life threatening tissue damage. Promising early clinical trials to use MSCs to treat GVHD patients still lack definitive, successful phase 3 trials. Notably, the Osiris Therapeutics Inc. sponsored phase 3 trial of MSC therapy for GVHD following hematopoietic stem cell transplantation did not meet its proposed endpoints across all ages but showed life-saving benefit in the pediatric patients.^127,128^ The results did lead to the first approvals for a culture-expanded MSC product for cell therapy against GVHD in Canada and New Zealand but did not achieve the studies endpoints necessary for US FDA approval. Instead, the MSC drug—“Prochymal” or “Remestemcel-L”—was made available in seven countries under the Expanded Access Program. Mesoblast Inc. acquired a license from Osiris to pursue culture-expanded MSCs in 2013 and the phase 3 trial for pediatric GVHD has recently completed enrollment with results expected soon. Several meta-analysis studies each comprising ~300 patients indicate the MSC treatments were effective in certain subpopulations but not all patients, and the reasons for this are unclear.^129–131^ However, given the diverse patient populations, varied MSC preparation methods, timing of first MSC infusion, dosing and heterogeneous pharmacological patient treatments, many possible improvements should be considered.

## Treating tissue injuries with MSCs

Mature adult tissues regularly perform maintenance and replace cells that have half-lives of hours to days, or from months to years, and in rare cases decades, e.g. brain neurons, chondrocytes, and cardiomyocytes where the cell half-life is on the order of ~50 years.^132^ When a tissue is damaged by trauma or disease, these regenerative repair processes may be accelerated, but this capacity diminishes with age, and each tissue ages somewhat differently.

Currently, a typical therapeutic dose of MSCs is 100 million cells and this number of packed cells occupies only ~400 μl. Most bodily injuries of this size are not a problem and clearly this “therapeutic” MSC dose is meant to initiate or augment a repair response from the body rather than serve as “cell for cell” replacement. For example, the adult heart is about the size of two hands clasped together and a “heart attack” may destroy a tissue volume similar to one, two or three fingers. Clearly, the current “therapeutic dose” of MSCs represents only a small portion of the total damaged cells in the tissue but this dose can produce clinically beneficial effects (see below). Further, although multiple dosing with MSCs is possible and many clinical trials now include this provision, it is well known that few transplanted MSCs engraft and survive and as few as <1% may be detectable later. This limited engraftment of transplanted cells is a major problem and not unique to MSCs, being well known in other cellular therapy fields including hematopoietic stem cell transplantation, CAR T-cell therapy, etc.

Most in vitro culture conditions do not prepare the MSCs for the in vivo setting, and there is little evidence for in vivo proliferation of delivered MSCs—likely due to their strong cell−cell contact inhibition of cell division and the lack of a ready “MSC-friendly” niche. Perhaps an exception to the rapid loss of transplanted MSCs is when they are implanted attached to a matrix such as for repairing a bone injury. Their increased survival when attached is likely due to intracellular signaling pathways, including focal adhesion signaling. Facilitating MSC homing to favorable sites of engraftment such as by improving MSC binding to sites of injury through drug pretreatment for attachment to ICAM-1 rich areas^133^ should improve in vivo survival. Additionally, most laboratories culture MSCs in atmospheric oxygen (20%)—out of convenience more than designing for cell optimization—but once implanted the MSCs must adapt quite quickly to much lower tissue oxygen levels. There are many studies that have used low oxygen cultivation (1−5% O_2_) of MSCs with good success and increased survival (see Pezzi et al.^134^ and references therein). Therefore, greater effort to prepare MSCs metabolically for the in vivo environment, including low oxygen cultivation, priming for glycolysis and cell attachment, may pay big dividends with respect to enhancing clinical efficacy.

Further, every tissue is complex and composed of several cell types, and the functional parenchyma of any tissue is accompanied by cells of the structural connective tissue. There are blood vessels (to deliver nutrients, remove wastes), sympathetic neurons (for central control), and lymphatic ducts (additional waste product removal) present in every cm^3^ of tissue with few exceptions. Due to a tissue’s metabolic needs, it is estimated that every cell in the body is no more than 2−3 cell diameters from a blood vessel, except in cartilage and cornea. Therefore, the repair of damaged functional tissue requires replacing several cell types, and, if using only MSCs for cell replacement therapy, requires the body to supply any missing cells. Although most studies use MSCs alone to attempt tissue repair/regeneration, most investigators agree that additional cell type(s) participate. For example, human endothelial colony forming cells (hECFCs) circulate in the peripheral blood and are capable of vasculogenesis, and when co-transplanted with MSCs, the hECFCs increase the engraftment and differentiation of MSCs in a PDGF-BB-dependent manner.^135^ Three-dimensional matrices and decellularized tissue scaffolds have also become a new proving ground for investigation of MSC potential and interaction with other cell types.^136,137^ More effort to understand the MSC interactions with endothelial progenitor cells, and epithelial cell types in various tissues, may provide important insight and opportunity for more effective tissue repair and clinical treatments.

## Clinical progress with MSCs for cardiac injuries, immunologic diseases and aging frailty

Over the past decade numerous advances have been made in the development of allogeneic MSCs as a therapy for a highly diverse group of diseases, including cardiac diseases. As described already, MSCs were considered a multipotential cell envisioned to differentiate into a limited repertoire of mesodermal tissues—bone, tendon, cartilage, muscle, and fat. However, it is now appreciated that MSCs produce many bioactive factors. This can provide a multiplexed approach and is likely effective for both post-infarct and nonischemic left ventricular failure. In this regard, human MSC delivery into the injured heart has demonstrated four potent mechanisms of action that work in concert: reduction of fibrosis, stimulation of neovascularization, immunomodulation, and stimulation of endogenous tissue regeneration.^138–140^

**Fig. 4:**
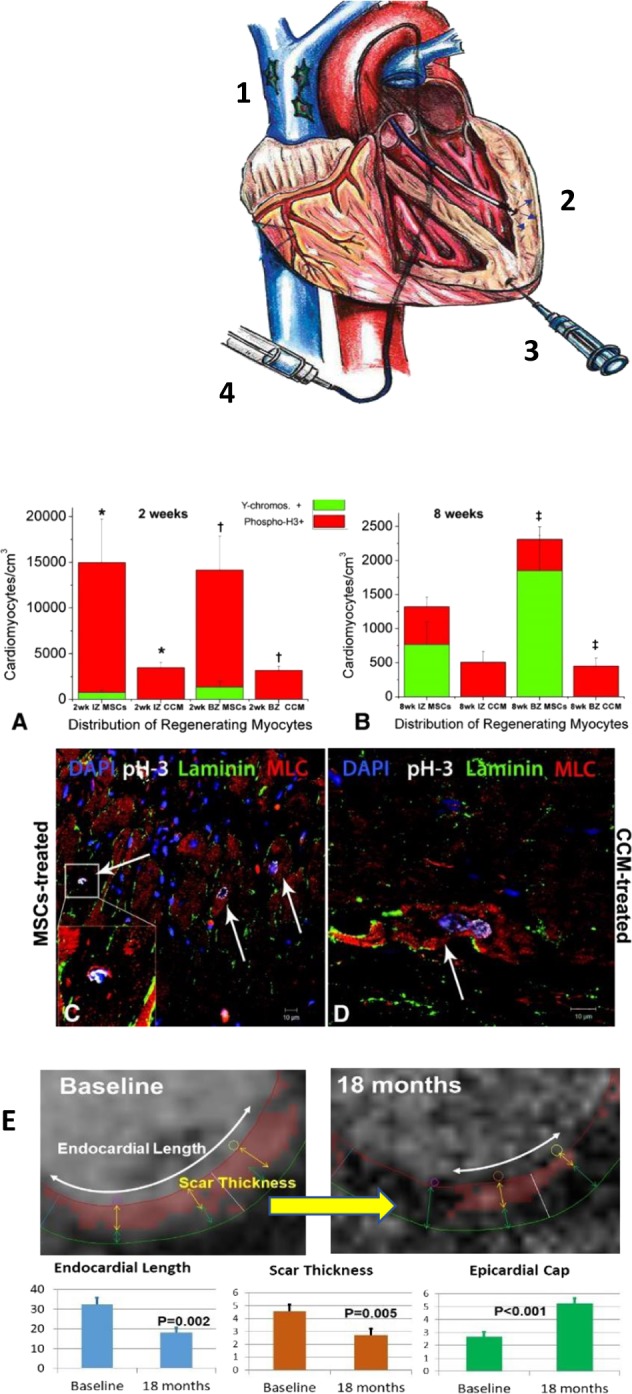
MSCs implanted in vivo in the infarcted left ventricle wall improve cardiac recovery in preclinical models and patient studies. A diagram of cellular therapy approaches tested with MSCs is shown. 1—Peripheral veinous infusion. 2—Endomyocardial delivery via injection catheter. 3—Direct myocardial injection during open chest surgery such as for coronary artery bypass grafting. 4—Delivery via intracoronary arteries. MSCs release anti-inflammatory factors and interact with endogenous cells to improve physiological outcome despite limited engraftment. Panels (**a**−**d**)^145^—**a** Porcine female heart receiving male allo-MSC injection show greater repair processes with active stimulation of endogenous cardiomyocyte cell-cycle activity (phospho H3 staining) which are associated with greater functional recovery. **b** Following direct injection of male MSCs near the infarct border, there are increased phospho-H3 detected at 8 weeks in the infarct (IZ) and border zones (BZ) compared to the remote zones (RZ) away from the infarct. The error bars indicate the mean ± SEM. **c**, **d** Immunohistology of data in (**a**) and (**b**). Results of clinical delivery of MSCs (**e**) are shown with MRI cross-section of hearts from patients receiving standard of care or standard of care plus MSCs in the PROMETHEUS trial.^141^ The MSC-treated hearts showed smaller infarcts at 12 and 18 months. The MSC-treated patients also had greater heart function (ejection fraction) and stamina (6 min walk test).^141^ The error bars indicate the mean ± SEM. Figures reproduced with permission of Kluwers Wolter/Circulation Research/Hare.^141,145^

These four combined actions are particularly powerful at avoiding the negative remodeling of organs damaged by ischemic injury. For example, post-myocardial infarction remodeling is the cause of substantial morbidity and mortality. The underlying driver of this disease process is ischemic injury to the heart leading to a loss of contractile cardiomyocytes and their replacement by a large area of fibrotic scar tissue. Numerous preclinical and clinical trials have demonstrated that injection of MSCs into the border zone between infarcted and viable cardiac tissue results in a powerful antifibrotic effect, reduced tissue injury and augmentation of viable and perfused tissue.^141–145^ The improved contractile cardiac muscle results predominantly from enhanced endogenous regeneration mechanisms rather than engraftment and differentiation of the injected MSCs.^146,147^ This conclusion derives from observations that relatively few MSCs are found engrafted at the site of injury relative to the degree of functional recovery, and that endogenous precursor cells and myocyte mitosis is upregulated with MSC treatment (Fig. 4). Recently, there is much interest in defining the molecular pathways and signaling modes for MSC activation of endogenous cell-cycling; for example, it has been shown that cell therapy may activate endogenous cardiac repair mechanisms by dual inactivation of the retinoblastoma and CDKN2a pathways.^148^

Also, the MSCs’ powerful immunomodulatory effects reduce levels of inflammatory cytokines such as TNFα at the injury site. As described earlier, the MSCs act by modulating inflammatory T-, B-, and NK-cell subsets, either at the injury site or by trafficking to the spleen to reduce its inflammatory responses, and/or lodging in the lungs and releasing soluble TSG-6 (see Fig. 3). The immuno-privileged and immunomodulatory MSCs appear safe for allogeneic therapy in the heart and the potent immunomodulatory properties of MSCs have also led to their widespread testing in immunologic disorders ranging from multiple sclerosis to aging frailty.^149,150^ Recently, several groups have become interested in repeat dosing regimens after cardiac infarct to bolster the effects of MSC therapy.^151–153^ Despite the MSCs’ diverse secretory repertoire, it is also apparent that MSCs themselves render cell autonomous effects through hetero-cellular coupling mechanisms including connexins in tissues such as the heart and spleen.^153^

Several exciting new approaches with MSCs are being tested to enhance therapeutic responses in cardiac damage. First, observations that MSCs interact with endogenous repair pathways led to the hypothesis that cell mixtures (i.e., MSCs plus cardiac stem cells (CSCs)) might enhance cardiac repair mechanisms (Fig. 4). This approach is borne out in animal models^146,147,151^ and presently being tested in an NHLBI clinical trial.^154^ Other strategies include seeding MSCs on tissue scaffolds to enhance their retention, and the repeat dosing regimen mentioned earlier. Together these approaches should enhance the effects of MSC therapy and hopefully be clinically relevant. While much work remains to understand the full mechanistic underpinnings and therapeutic potential of MSCs in the heart, the clinical testing to date provides an important aspect to cardiac medicine where living cells become a key therapeutic approach.

## Optimizing MSCs for therapeutic purposes: tuning their output

As outlined above, MSCs can provide therapy for several clinical situations but the MSCs may exhibit different functional properties depending on how they are produced, handled and administered. The clinical benefits of MSC treatment involve the modulation of the immune system and the improved functionality of the damaged tissue. However, not all patients respond and the MSCs may provide potent effects in 40–50% of patients, meaning there is much more to understand about MSC therapy in patients. This may not be so different from the published clinical trial results from any cellular therapy under development but deserves careful investigation. The nonresponders among patients receiving MSCs appear to be a reflection of a combination of factors; the MSC production method for therapy, the cells’ metabolic activity, the delivered dose, the stage of the disease, and the status and/or genetic receptivity of the patient.^155,156^ Given this complexity, it has become apparent to many researchers that the MSCs used for therapy must be carefully produced and properly “tuned” for the intended therapy. The tuned MSCs should be optimized for the required medicinal response and for the patients’ capacity to respond, as best this is understood. For example, currently, a single production method may be used to produce doses of MSCs administered for treatment of conditions as diverse as graft-versus-host disease, acute myocardial infarct or lung injury. Clearly, the same MSC production process is not optimized (i.e., tuned) to provide the “best” therapeutic benefit for very different clinical indications. Therefore, while the MSC field has often adopted a reliance on the MSCs “knowing what to do”, today a more sophisticated approach is needed to enhance the cell production, delivery and efficacy of MSC-based therapies.

Optimizing the production of MSCs for a particular medical indication should improve outcomes, and this may involve identifying the marrow donors whose culture-expanded MSCs exhibit an optimized response in a relevant assay that addresses the clinical situation, such as the aforementioned CLIP assay.^67^ For a current example, the sponsors Case Western Reserve University, the National Center for Regenerative Medicine and University Hospitals Rainbow Babies and Children’s Hospital in their investigator-sponsored Phase I clinical trial to treat individuals with cystic fibrosis (CF) who have lung infections, have chosen MSC donors based on selected criteria. In this case, donor marrow aspirates were used to isolate and culture MSCs and then each donor’s MSCs were tested by exposure to *Pseudomonas aeruginosa* or *Staphylococcu*s bacteria in culture and the culture medium analyzed for the antibiotic protein LL37 and inflammatory mediators. From this test, one donor’s MSCs had a very high expression of these bioactive molecules compared to all the other donor MSCs. Thus, from the in vitro assay, an MSC donor was selected that exhibited an optimized response to the bacteria that are medically relevant for CF-patients with lung infections. The clinical trial is underway and should establish safety and, hopefully, some efficacy of these selected MSCs. Outcomes of such trials are critical to assess the nature and type of surrogate potency assays that may be needed to predict efficacy of MSC-based therapies in patients.

To prepare MSCs for a harsh in vivo environment, selected agents may be added to the in vitro MSC-media to sensitize and adapt the MSCs to the destructive microenvironment that they next encounter. For example, in the case of patients newly diagnosed with rheumatoid arthritis (RA), the production of MSCs for this therapy should expose cells to a strong inflammatory mediator like IL-1. MSCs exposed to IL-1 mount an anti-inflammatory response within 24−48 h that can be monitored by analyzing the culture medium and identifying the most effective donor MSCs to provide greater treatment efficacy.^157^ Preconditioning the MSCs in culture to an inflammatory environment will optimize the MSCs to the microenvironment they will experience when infused into an RA patient. These approaches represent progress toward achieving therapies tailored for specific disease indications, and represent a form of “personalized medicine”, that could potentially result in cost−benefit returns and may accelerate the FDA approval process. An important scientific question is how to optimize the responsiveness of MSCs for greater therapeutic effects and clinical benefit. If the untuned MSCs provide potent effects in about half of all patients, perhaps tuned MSCs and selected patients will have much better outcomes and provide a rational basis for further improvements. In this regard, current production MSCs are also a fine starting point for further manipulation to enhance their multilineage potential to not only tune them but guide them towards desired cell types as seen in guided cardiopoiesis^17^ or induction of hepatocyte function^158^ by physical as well as chemical stimulation. Such optimization requires unique and collegial interactions between academics, industry, doctors, patients and administrators.

## The MSC process is the MSC product

How can the MSC in vitro expansion process be refined to produce the best therapeutic MSCs in large amounts in a reproducible and reliable manner? For MSCs, the “process is the product” refers to the quality by design concept that all critical sources of variability are identified and explained, and the product quality attributes can be accurately and reliably predicted over the design space established for the materials used, the process parameters, the manufacturing environment and any other pertinent conditions. When the process is accurate and followed precisely, the product will be the same each time. If the product does not meet its release criteria, a review of the procedures is needed to understand what is happening at critical steps, and thereby seek continuous improvement.^159^ This is where we are today. Listed in Table 2 are the topics and areas for improvement for MSC studies. With MSCs and their variable nature—a feature not a flaw—it is necessary to control each step in their cultivation for research and therapeutic use in order to have reproducible results. Critical steps along the cell production process should have an assay that can evaluate progress along the desired path. For the academic laboratory, assay reproducibility is essential, and the notion of continuous improvement should be embraced, while for the cell therapy facility and commercial producers this is a primary and legal responsibility. As discussed earlier, the MSC isolation, establishment in culture, and final expansion are complicated by the clonal expansion/extinction that needs further investigation. Still, we believe there are process steps that can be controlled and consistently met to achieve reproducible results across different laboratories and geographic locations that will result in reproducible MSC cell therapy outcomes. However, this is not an automatic endpoint from the current knowledge base and will require vigilance, persistent effort, and collegial communications.

**Table 2:** A number of study areas are suggested where new results could improve the understanding of MSC basic science and/or clinical therapies.

Challenges in the field	Opportunities/solutions
Defining the “MSC” and specific MSC populations for therapy	Reconcile lineage tracing/genetic results with phenotypic/functional studies of cultured MSCs
Tissue-resident MSCs	Omics-based approaches, rigorous (and tissue relevant) functional testing to define similarities and differences
Decrease in in vivo MSCs with age	Augment with ex vivo produced MSCs
Intra/inter-population heterogeneity	Longitudinal culture assays, genetic tagging, bar coding
Use of autologous vs. allogeneic MSCs	Test in parallel in vitro and in vivo, disease relevance
MSCs for tissue replacement	Fate priming w/cytokines and culture conditions, substrates (rigid or flexible, etc.), smart scaffolds, engineered tissue
MSCs into injured tissue	Assays for altered expression of factors and exosome contents of in vitro cultured MSCs placed in the in vivo and injured tissue setting
Lack of engraftment	Preconditioning for in vivo metabolism and hypoxia to prevent apoptosis; scaffolds as delivery vehicles, recovery from freezing prior to infusion
Dosing regimens	Single bolus, or repeated or escalating doses
Delivery methods	Direct, local, or systemic—optimize for tissue and disease type
Disease-specific treatments	“Tune” MSCs using biologics, select for defining traits via potency assays, CLIP scale
Cell therapy release assays	Not universal but designed for the specific disease, target tissue, and patient population

## Conclusions

We have discussed important aspects of MSCs and MSC-like cells based on our understanding of the field’s nearly three decades of development, the current state of adult stem/progenitor cell science, and cellular therapy technologies. The MSCs continue to undergo testing in a broad spectrum of clinical trials, some of which have strong support from animal model studies, but other areas too where the patient need is great and some MSC attributes suggest they would provide benefit. We have pointed out in Table 2 areas where further studies could advance the understanding and the utility of MSCs. The MSCs have properties not found in other stem/progenitor cells and these can be harnessed in many ways as the progress to date demonstrates. In retrospect, MSCs may have started as “a riddle wrapped in a mystery, inside an enigma” (W. Churchill on a separate topic during WWII), but years of research have shown that MSCs are a powerful cellular entity that interacts with their immediate surroundings and neighboring cells to provide cell-based responses that can be therapeutic. There remains much to be gained in terms of scientific knowledge and clinical benefit as the complex biology and therapeutic potential of MSCs are more fully understood.

### Reporting summary

Further information on research design is available in the Nature Research Reporting Summary linked to this article.

## Supplementary information

Reporting Summary Checklist
